# Protective Effect of Cast‐Off Skin of *Cicadidae Periostracum* Water Extract in a Radiation‐Induced Testicular Injury Mice Model

**DOI:** 10.1002/fsn3.70198

**Published:** 2025-04-24

**Authors:** Yun‐Soo Seo, Seung Mok Ryu, Jun Lee, Huiyeong Jeong, Goya Choi, Byeong Cheol Moon, Je‐Oh Lim, Hyeon‐Hwa Nam, Joong‐Sun Kim, Sueun Lee

**Affiliations:** ^1^ Herbal Medicine Resources Research Center Korea Institute of Oriental Medicine Naju Republic of Korea; ^2^ College of Veterinary Medicine and BK21 Plus Project Team Chonnam National University Gwangju Republic of Korea; ^3^ Department of Crop Science & Biotechnology Jeonbuk National University Jeonju Republic of Korea

**Keywords:** antioxidation, *Cicadidae Periostracum*, radiation therapy, testicular injury

## Abstract

Radiation therapy has been used to treat cancer; however, the associated DNA damage and adverse effects in the surrounding tissues remain major concerns. In radiation injury, several natural remedies can effectively relieve symptoms with minimal side effects. *Cicadidae Periostracum* (CP, also called “Sun‐Tae”) is the cast‐off shell of *Cryptotympana pustulata* Fabricius and has been traditionally used for several pharmacological actions. This study investigated the protective effects of CP water extract (CPE) on irradiated testicular tissue and spermatogenesis. The chemical constituents of CPE were analyzed using a high‐performance liquid chromatography system equipped with a mass detector and were determined to be dimeric *N*‐acetyl dopamine derivatives. The testes and epididymal tissues of male C57BL/6 mice orally pretreated with CPE (25 and 50 mg/kg) 24 h and 15 min before radiation exposure (5 Gy) were collected for analysis. CPE pretreatment ameliorated radiation‐induced apoptosis in the testes and inhibited weight loss in the epididymis compared to those of the irradiated group. Treatment with high‐dose CPE prevented the radiation‐induced decrease in the epithelial height of seminiferous tubules. In addition, the morphology, motility, and number of damaged sperm cells following radiation exposure were alleviated by CPE treatment. Furthermore, the dose‐dependent antioxidative activity of CPE was confirmed in 2,2‐diphenyl‐1‐picrylhydrazyl and 2,2′‐azinobis‐(3‐ethylbenzothiazoline‐6‐sulphonic acid) radical scavenging assays, and reactive oxygen species generation by hydrogen peroxide was reduced by CPE treatment in vitro. Our findings suggest that pretreatment with CPE can ameliorate testicular tissue damage and sperm degeneration via the antioxidative properties of CPE in radiation‐injured testis tissue.

## Graphical abstract

*Cicadidae Periostracum* water extract (CPE) pretreatment improved radiation‐induced testicular damage, including apoptosis and decrease of epididymis weight, epithelial height, sperm motility, and sperm number. Two dimeric *N*‐acetyl dopamine derivatives were isolated from CPE, and the antioxidative activity of CPE was confirmed in radical scavenging and 2,7‐dichlorodihydrofluorescein diacetate assays. Our findings suggest that CPE pretreatment can ameliorate testicular tissue damage and sperm degeneration via the antioxidative properties of CPE in radiation‐injured testis tissue.

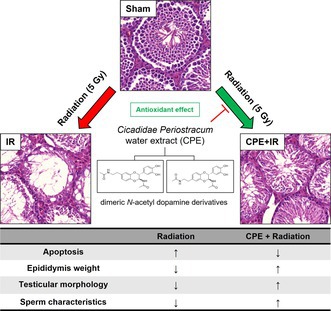

## 1 Introduction

Radiotherapy, widely used in cancer treatment (Borek 2004), damages the DNA of cancer cells directly through ionization or indirectly via the generation of reactive oxygen species (Berkey 2010). However, side effects can also impact surrounding tissues (Bentzen 2006). Several tissues, including the testes, bone marrow, and colon, are sensitive to radiation (Majeed and Gupta 2025). The testes, particularly, are directly or more frequently exposed to scattered radiation during therapy in nearby tissues, including the prostate and penis (Okada and Fujisawa 2019). Exposure to even extremely low doses of radiation can damage spermatogenic cells in the testes; thus, temporary or permanent infertility is a common post‐irradiation complication (Kovacs and Stern 1999). Although effective cancer therapy is of utmost importance, potential gonadal damage may cause great distress, particularly in patients of reproductive age (Dillon and Gracia 2012). Therefore, developing nutraceuticals and pharmaceuticals that minimize radiation‐induced testicular injury remains essential.

Owing to their low toxicity and easy accessibility, traditional medicines have been widely applied to treat various diseases (Tiwari et al. 2020). *Cicadidae Periostracum* (CP), a cast‐off shell of *Cryptotympana pustulata* Fabricius called “chantui” in Chinese medicine (Luo et al. 2022), is referred to as “Sun‐Tae” described in the Chung‐bu category of an ancient Korean medical book named *Dongui Bogam* (Lim et al. 2019). Research on electronic databases using the Oriental Medicine Advanced Searching Integrated System has shown that *Cicadidae Periostracum* (CP) has been traditionally used to treat spasms, tetanus, sore throat, colds, and pruritus. Recent studies have demonstrated various pharmacological effects of CP, including the suppression of inflammation (Park et al. 2021), antibacterial activity (Wang et al. 2010), and modulation of atopic dermatitis (Nam et al. 2018). Furthermore, the components isolated from the CP water extract (CPE), including *N*‐acetyl dopamine dimers (Thapa, Katila, Choi, Han, et al. 2021) and suntamide A (Thapa, Katila, Choi, Choi, et al. 2021), exert various biological effects. However, scientific evidence of the protective effects of CP against radiation‐induced oxidative stress and male gonadal damage remains insufficient. Therefore, this study aimed to determine whether CP protects against radiation‐induced testicular dysfunction by estimating various parameters related to testicular injury.

## 2 Materials and Methods

### 2.1 Preparation of CPE

CP was acquired from Kwangmyungdang Medicinal Herb (Ulsan, Korea) and confirmed macroscopically by Dr. Goya Choi (Herbal Medicine Resources Research Center, Korea Institute of Oriental Medicine, Naju, Korea). CPE was prepared as follows (Lim et al. 2019): Briefly, CP was extracted with distilled water for 3 h under reflux (100°C ± 2°C). The extract was filtered, evaporated in a rotary air vacuum evaporator, and lyophilized (yield: 6.30%). The original CP (medicinal resource ID: 2‐18‐0119) and CPE (3‐18‐0038) were stored at −20°C in the Herbal Medicine Resources Research Center.

### 2.2 High‐Performance Liquid Chromatography System (HPLC) Equipped With a Single Mass Spectrometer (MS) Analysis of CPE

The chemical constituent analysis of CPE was performed using a HPLC (e2695, Waters, Milford, MA, USA) equipped with a single MS (Acquity QDa, Waters). Chromatographic separation was performed using an Xbridge BEH C_18_ column (Waters, 4.6 × 150 mm, 3.5 μm). The mobile phases comprised 0.1% formic acid in water (A) and 0.1% formic acid in acetonitrile (B) implemented in the gradient condition: 5%–100% (B) for 0–7 min; 100% isocratic (B) for 7–8.5 min; 100%–5% (B) for 8.5–8.6 min; 5% isocratic (B) for 8.6–10 min with a column flow rate of 1.0 mL/min. The chemical constituents of CPE were identified based on the MS spectra of previously isolated compounds at the Korea Institute of Oriental Medicine.

### 2.3 Animals

Male C57BL/6 mice, 7‐ to 8‐week‐old, were obtained from DooYeol Biotech (Seoul, Korea) and were housed in a room maintained at a temperature of 23°C ± 2°C (humidity of 50% ± 5%), artificial lighting from 08:00 to 20:00, and 13–18 air changes per hour. Mice were provided food ad libitum and cared for in accordance with the National Institutes of Health Guide for the Care and Use of Laboratory Animals. All animal protocols were approved by the Institutional Animal Care and Use Committee of the Korea Institute of Oriental Medicine (KIOM 20‐002, January 2020).

### 2.4 Radiation Exposure

After anesthetizing the mice with 85 mg/kg alfaxalone (Alfaxan; Careside, Gyeonggi‐do, Korea) and 10 mg/kg xylazine (Rompun; Bayer Korea, Seoul, Korea), single fractions of 5 Gy were delivered at a specific depth through a distal pelvic field (2 cm × 2 cm). The testes were completely irradiated using ^60^Co gamma rays (Elekta, Stockholm, Sweden) at a dose rate of 3.8 Gy/min. The sham‐irradiated group was treated in the same manner as the irradiated (IR) group.

### 2.5 Experimental Schedules

We conducted two sets of experiments with different euthanasia timepoints after irradiation. The mice were randomly divided into five groups for each experimental set. Radiation (5 Gy) was applied to the pelvis in the IR group. CPE (25 and 50 mg/kg) dissolved in phosphate‐buffered saline (PBS) was orally administered 24 h and 15 min before irradiation. In the vehicle groups, including the sham and IR groups, PBS was administered instead of CPE. The mice were euthanized 12 h (*n* = five per group) and 35 days (*n* = five per group) after irradiation for the first and the second set of experiments, respectively.

### 2.6 Apoptosis Assay

The anti‐apoptotic effect of CPE was investigated in mice euthanized 12 h after irradiation; based on a previous study, radiation‐induced apoptosis peaks at 12 h post‐irradiation (Lee et al. 2008). The terminal deoxynucleotidyl transferase dUTP‐biotin nick end labeling (TUNEL) method was used for immunohistochemical staining using a commercial kit (Abcam, Cambridge, MA, USA). Tissue samples were prepared as sections along the long axis. Two sections from each group were examined, and the TUNEL‐positive cells in each seminiferous tubule were counted under a microscope as previously described (Kim et al. 2011, 2012).

### 2.7 Histological Analysis

In mice, one cycle of spermatogenesis takes approximately 35 days (Oakberg 1956). Therefore, in mice euthanized 35 days after irradiation, both the testes and epididymal tissues were dissected from the surrounding tissues, and their average weights were measured. The right testis was fixed using Bouin's fluid solution for 2 days and embedded in paraffin. Histological parameters were investigated using hematoxylin and eosin staining. The epithelial height and diameter of seminiferous tubules were measured using an ocular micrometer. Approximately 30 round tubules were measured from each mouse, excluding those at the edge of the testis. The epithelial height of 30 tubules was calculated for each mouse. The mean values were used to calculate the epithelial height of each group.

### 2.8 Sperm Evaluation

Sperm count and abnormality evaluations were conducted in the right caudal epididymis of mice euthanized 35 days (i.e., spermatogenesis cycle) post‐irradiation to assess the effect of the radiation or CPE treatment on testicular functions. The tissue was homogenized for 30 s in saline (0.5 mL). The aliquot of the homogenized sample (10 μL) was diluted in the dilution solution (0.25% trypan blue, 5% NaHCO_3_, and 0.35% formalin), and the sperm cells were counted using a hemocytometer. After mixing the sperm suspension with 1% eosin Y, the samples were smeared on a glass slide and evaluated by examining the head and tail defects of 400 spermatozoa per mouse under a light microscope at 400× magnification. Abnormal sperm morphology was confirmed as previously described (Kim et al. 2011, 2012). Amorphous sperm and deformed sperm, evident from the lack of a head hook, a folded tail, and a double tail were counted. The motility assay was performed as previously described (Nam et al. 2022). Briefly, the left caudal epididymis was placed in 100 μL of 5 mg/mL bovine serum albumin (BSA) and minced using scissors. The sample was incubated for 10 min at 37°C, and 20 μL was dispensed onto a slide for motility analysis. The number of mobile sperm was determined by counting all motile cells, and 100 sperms were counted. Sperm motility was assessed in semen samples from the same mouse (Kim et al. 2011).

### 2.9 2,2‐Diphenyl‐1‐Picrylhydrazyl (DPPH) Radical Scavenging Assay

100 μL of CPE (4, 20, 100, 500, and 2500 mg/mL; *n* = four per group) was mixed with 100 μL of DPPH solution in MeOH and reacted for 30 min at room temperature in the dark. The absorbance was measured at 515 nm using a microplate reader (SpectraMax i3X; Molecular Devices, San Jose, CA, USA). The percentage inhibition of the DPPH radicals was calculated as follows:

$$\begin{array}{c} \text{DPPH scavenging activity}\;\left(\%\right)\\ =\left(1-\left(A_{\text{sample}}-A_{\text{blank}}\right)/\left(A_{\text{control}}-A_{\text{blank}}\right)\right)\times 100 \end{array}$$

### 2.10 2,2′‐Azinobis‐(3‐Ethylbenzothiazoline‐6‐Sulphonic Acid) (ABTS) Radical Scavenging Assay

The preformed radical monocation of ABTS˙ + is generated by oxidation of 7 mM ABTS (≥ 98%, from Sigma‐Aldrich, South Korea) with 2.5 mM potassium persulfate (≥ 99%, from Sigma‐Aldrich, South Korea) at the ratio of 1:1. After 10 min, from the mixture of 20 μL of CPE (4, 20, 100, 500 and 2500 mg/mL; *n* = four per group) and 180 μL of diluted ABTS + solution (Abs_734nm_ = 0.7), the absorbance was measured at 734 nm using a SpectraMax i3x microplate spectrophotometer (Molecular devices). The percentage of ABTS radical inhibition was calculated as follows:

$$\begin{array}{c} \text{ABTS inhibition rate}\;\left(\%\right)\\ =\left(1-\left(A_{\text{sample}}-A_{\text{blank}}\right)/\left(A_{\text{control}}-A_{\text{blank}}\right)\right)\times 100 \end{array}$$

### 2.11 2,7‐Dichlorodihydrofluorescein Diacetate (DCFDA) Assay

The DCFDA assay was used to measure the effect of CPE on reactive oxygen species (ROS) production. HeLa cells were treated with CPE (5, 10, 50, and 100 μg/mL) and 1 mM hydrogen peroxide (H_2_O_2_) separately and incubated for 24 h. After incubation, cells were washed with PBS, and 100 μM DCFDA was treated in each well for 30 min at 37°C. Fluorescence intensity was measured using a fluorescence plate reader (Paradigm; Beckman Coulter, Brea, CA, USA) at excitation and emission wavelengths of 485 and 520 nm, respectively, after washing DCFDA with PBS.

### 2.12 Statistical Analyses

Data were analyzed using one‐way analysis of variance followed by a Student–Newman–Keuls post hoc test for multiple comparisons. The data are presented as mean ± standard error of the mean (SEM), and a *p*‐value of < 0.05 was considered significant.

## 3 Results

### 3.1 Chemical Constituent Analysis of CPE

**FIGURE 1:**
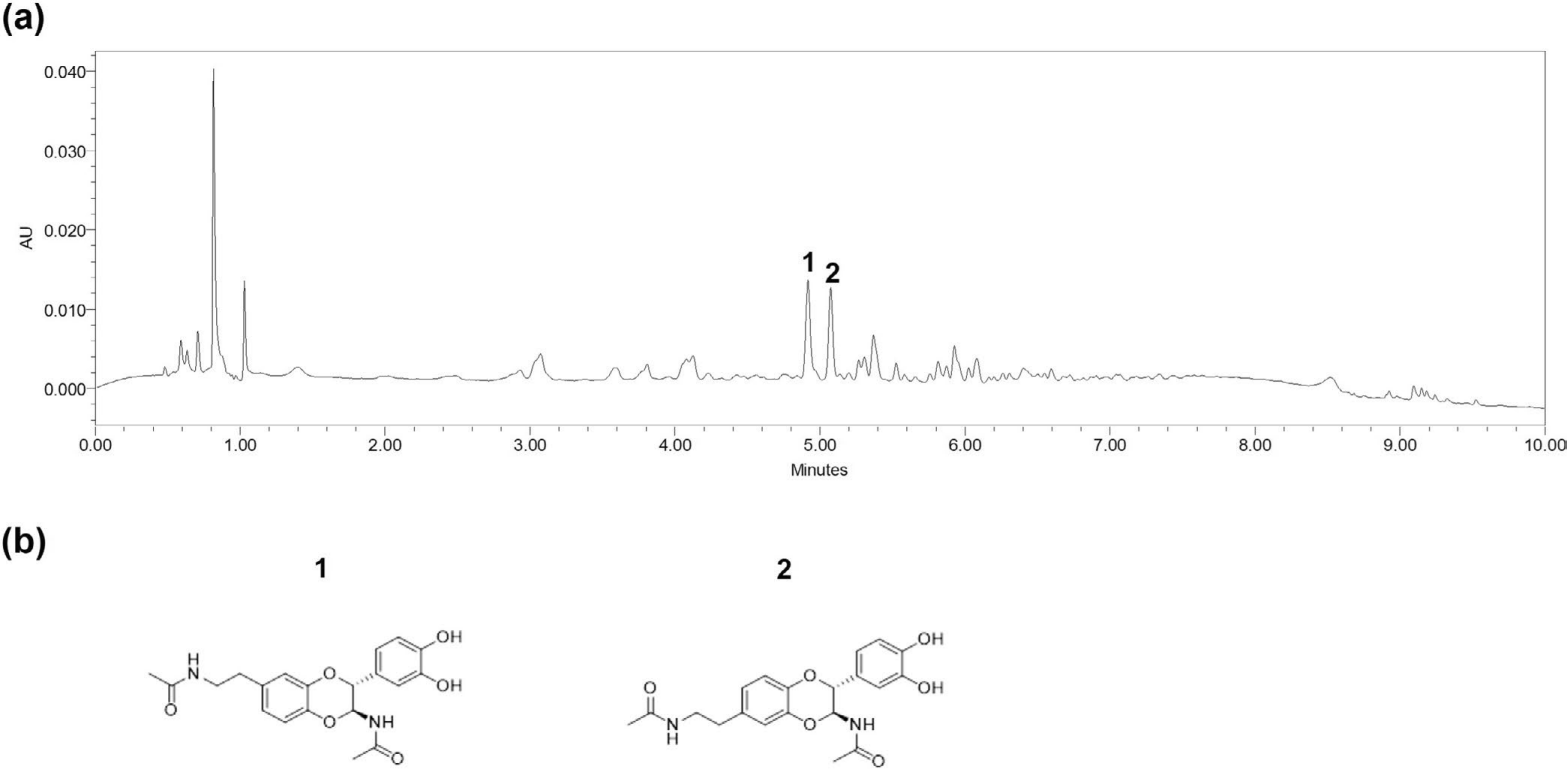
Chemical constituent analysis of CPE using HPLC‐MS. (a) UV chromatogram of CPE at 254 nm. (b) Chemical structure of each compound.

HPLC‐MS was used to analyze the chemical constituents in CPE. Compared with standard compounds under the same analysis conditions, the components extracted from CPE were dimeric *N*‐acetyl dopamine derivatives such as (2*R*,3*S*)‐2‐(3′,4′‐dihydroxyphenyl)‐3‐acetylamino‐7‐(*N*‐acetyl‐2″‐aminoethyl)‐1,4‐benzodioxane (**1**) and (2*R*,3*S*)‐2‐(3′,4′‐dihydroxyphenyl)‐3‐acetylamino‐6‐(*N*‐acetyl‐2″‐aminoethyl)‐1,4‐benzodioxane (**2**) (Lee et al. 2017) (Figure 1, Table 1).

**TABLE 1:** Major compounds identified using high‐performance liquid chromatography/mass spectrometry (HPLC‐QDa) and their contents in CPE.

No	Compound	Retention time (min)	m/z^a^	m/z^b^
**1**	(2*R*, 3*S*)‐2‐(3′,4′‐dihydroxyphenyl)‐3‐acetylamino‐7‐(*N*‐acetyl‐2″‐aminoethyl)‐1,4‐benzodioxane	4.91	385 [M—H]^−^	387 [M + H]^+^
**2**	(2*R*, 3*S*)‐2‐(3′,4′‐dihydroxyphenyl)‐3‐acetylamino‐6‐(*N*‐acetyl‐2″‐aminoethyl)‐1,4‐benzodioxane	5.10	385 [M—H]^−^	387 [M + H]^+^

^a^ Negative Ion mode.

^b^ Positive Ion mode.

### 3.2 Effect of CPE Pretreatment on Radiation‐Induced Apoptosis in Mouse Testis

**FIGURE 2:**
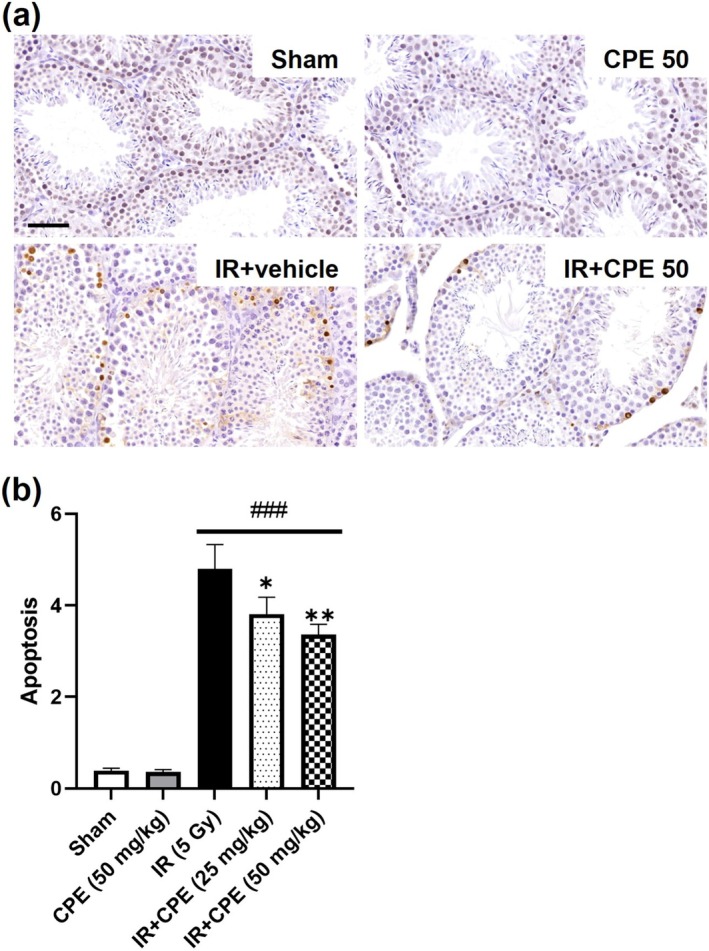
Protective effect of CPE pretreatment on apoptotic cell death in the testis following pelvic irradiation (5 Gy). The terminal deoxynucleotidyl transferase dUTP‐biotin nick end labeling (TUNEL) assay was performed 12 h post‐irradiation. Representative images (×400) show TUNEL‐stained sections of each group (a). Graphs show the number of TUNEL‐positive cells in each seminiferous tubule (b). Data are reported as the mean ± SEM (five mice in each group). ^###^ *p* < 0.001 versus sham group, *p < 0.05, and **p < 0.01 versus IR group. Scale bars = 50 μm. IR, pelvic irradiation; CPE, water extract of *Cicadidae Periostracum*; IR + CPE, mice pretreated with 25 or 50 mg/kg CPE before irradiation.

The effect of CPE on apoptosis was determined by counting the number of TUNEL‐positive nuclei in the testis tissue 12 h after radiation exposure. Figure 2 shows that the number of apoptotic cells per seminiferous tubule was significantly higher in the IR group than in the sham group (*p* < 0.0001). Treatment with CPE alone did not influence apoptosis (*p* = 0.9506), but a significant decrease in apoptotic nuclei was confirmed in the CPE (25 and 50 mg/kg)‐treated IR group compared with the IR group (*p* = 0.0347 and *p* = 0.0099, respectively). The number of apoptotic cells in the high‐dose CPE‐treated group before irradiation was slightly improved compared to that in the low‐dose CPE‐treated group, although the difference was not significant (*p* = 0.3216).

### 3.3 Effect of CPE Pretreatment on the Testis and Epididymis Weights Following Radiation Exposure

**FIGURE 3:**
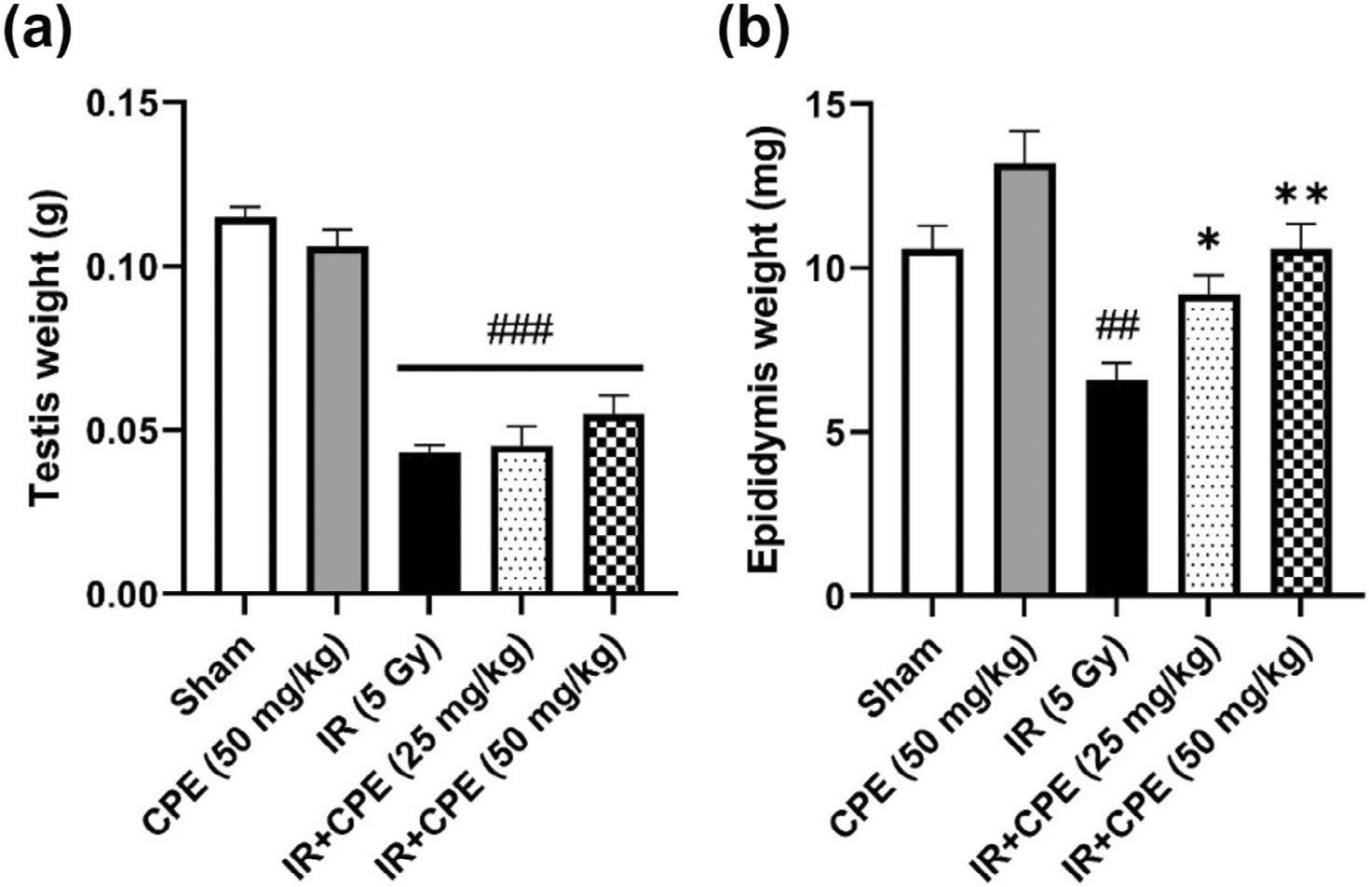
Protective effect of CPE pretreatment on testis (a) and epididymis tissue weight (b) 35 days post‐pelvic irradiation (5 Gy). Data are reported as the mean ± SEM (five mice in each group). ^##^ *p* < 0.01, and ^###^ *p* < 0.001 versus sham group, *p < 0.05 and **p < 0.01 versus IR group. IR, pelvic irradiation; CPE, water extract of *Cicadidae Periostracum*; IR + CPE, mice pretreated with 25 or 50 mg/kg CPE before irradiation.

We measured the weights of the testes and epididymis to confirm the effect of CPE pretreatment on tissues following 35 days after radiation exposure (5 Gy) (Figure 3). Only CPE pretreatment (50 mg/kg) did not affect testis weight (*p* = 0.1832), but slightly increased epididymal weight (*p* = 0.0461) compared with the sham group. The weights of the testis and epididymis were significantly decreased in the IR group compared with those in the sham group (*p* < 0.0001 and *p* = 0.0022, respectively). Although there was no significant difference, CPE pretreatment (50 mg/kg) showed a slight protective effect against radiation‐induced testis weight loss (*p* = 0.1895). CPE pretreatment (25 and 50 mg/kg) before radiation significantly alleviated the epididymis weight loss compared to that observed in the IR group (*p* = 0.0183 and *p* = 0.0040, respectively), and among groups, the high‐dose CPE‐pretreated group had an epididymis weight similar to that in the sham group. The high‐dose CPE treatment before radiation slightly improved the weights of the testis and epididymis compared with those in the low‐dose CPE‐treated group before radiation, although the difference was not significant (*p* > 0.9999 and *p* = 0.3681, respectively).

### 3.4 Effect of CPE Pretreatment on the Testicular Morphology Following Radiation Exposure

**FIGURE 4:**
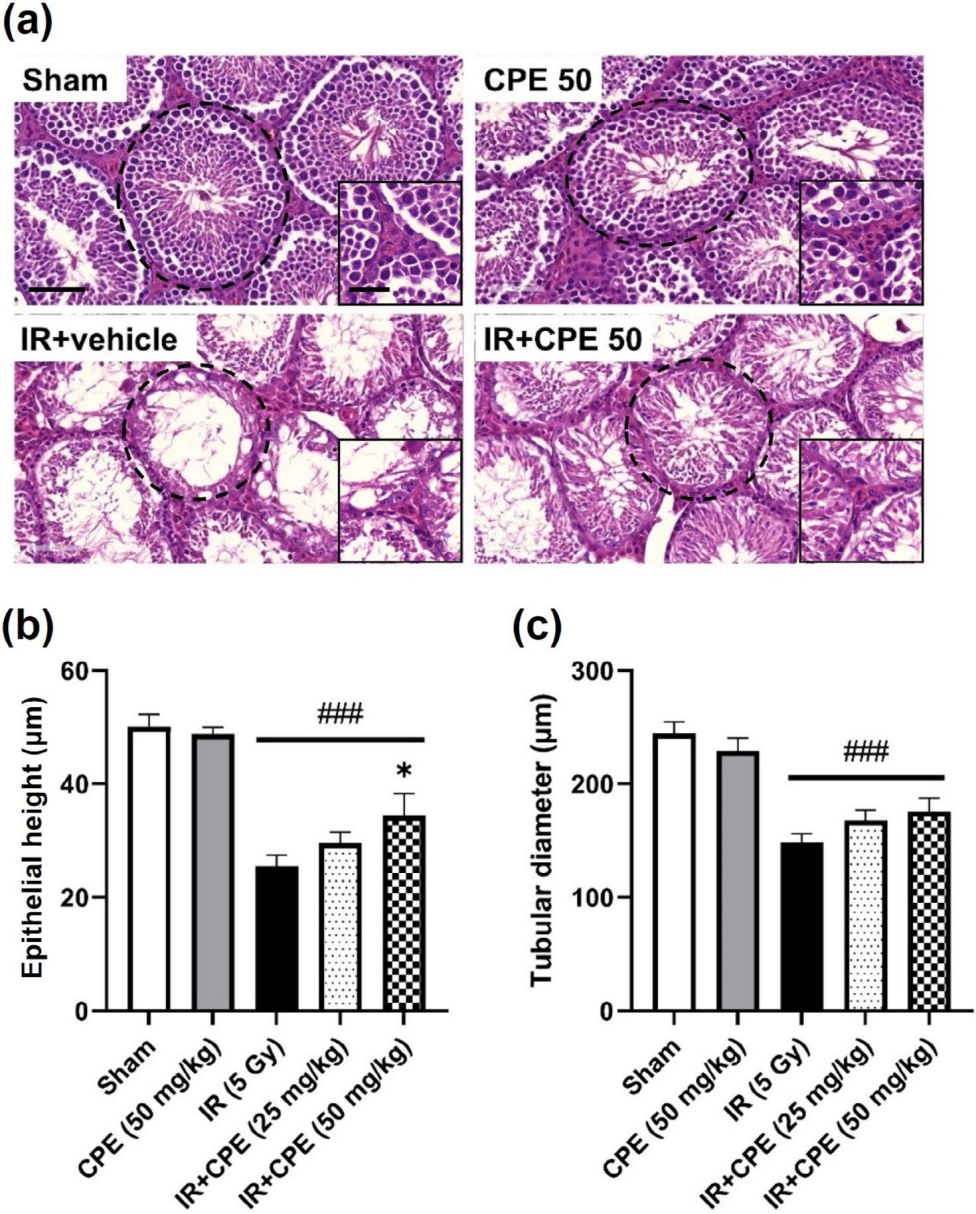
Protective effect of CPE pretreatment on histopathology of the testis tissue 35 days post‐pelvic irradiation (5 Gy). Representative images (×400) show H&E‐stained sections for each group (a). The dotted line and inserted magnified photo represent the seminiferous tubule and the interstitial space of the testis, respectively, in each group. Graphs show the histological features of the testicular epithelial height (b) and seminiferous tubular diameter (c). Data are reported as the mean ± SEM (five mice in each group). ^###^ *p* < 0.001 versus sham group, *p < 0.05 versus IR group. Scale bars = 70 and 35 μm for low and high magnification, respectively. IR, pelvic irradiation; CPE, water extract of *Cicadidae Periostracum*; IR + CPE, mice pretreated with 25 or 50 mg/kg CPE before irradiation; H&E, hematoxylin and eosin.

We examined the histological changes in the testes induced by radiation exposure or CPE pretreatment 35 days post‐irradiation. As shown in Figure 4a, the sham group showed normal testicular architecture and seminiferous tubular morphology, and the CPE pretreatment (50 mg/kg) group showed a similar morphology to the sham group. Radiation exposure damaged the testicular architecture, including cell loss and seminiferous tubular atrophy. However, CPE pretreatment (50 mg/kg) alleviated radiation‐induced morphological changes. The structure of the testicular interstitial space was not significantly affected by the radiation.

Morphological damage was quantified by measuring the testicular epithelial height and seminiferous tubular diameter at 35 days (Figure 4b,c). The sham and CPE (50 mg/kg)‐treated groups showed similar testicular morphology parameters, whereas the epithelial height and the tubular diameter of the IR group were substantially decreased compared to those in the sham group (both *p* < 0.0001). However, CPE pretreatment (25 and 50 mg/kg) before radiation exposure had a dose‐dependent radiation‐protective effect on the epithelial height (*p* = 0.2499 and *p* = 0.0410, respectively) and seminiferous tubule diameter (*p* > 0.9999 and *p* = 0.1653, respectively), although the significance was only detected in the epithelial height index. There were no significant differences in both indexes between the CPE 25 mg/kg‐ and 50 mg/kg‐pretreated groups before irradiation (*p* = 0.1649 and *p* > 0.9999, respectively).

### 3.5 Influence of CPE Pretreatment on Sperm Characteristics Following Radiation Exposure

**FIGURE 5:**
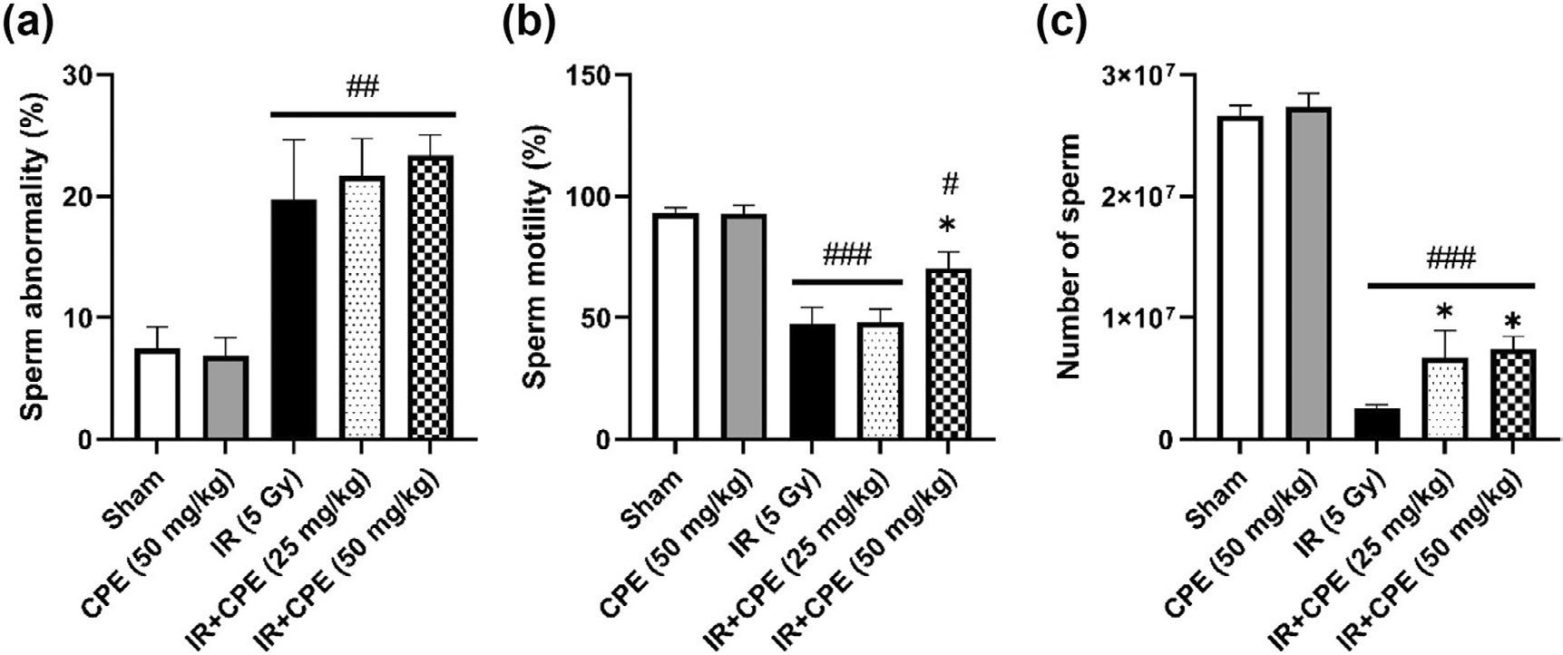
Effect of CPE pretreatment on sperm characteristics in the testis 35 days post‐pelvic irradiation (5 Gy). Graphs showing sperm abnormality (%) (a), sperm mobility (%) (b), and number of sperm (c). Data are reported as the mean ± SEM (five mice in each group). ^#^ *p* < 0.05, ^##^ *p* < 0.01, and ^###^ *p* < 0.001 versus sham group, *p < 0.05 versus IR group. IR, pelvic irradiation; CPE, water extract of *Cicadidae Periostracum*; IR + CPE, mice pretreated with 25 or 50 mg/kg CPE before irradiation.

The effect of CPE pretreatment on the sperm of the irradiated testis tissue was confirmed by observing sperm abnormalities, sperm mobility, and sperm number 35 days post‐irradiation. Sperm abnormalities in the IR group significantly increased compared with those in the sham group (*p* = 0.0077; Figure 5a). CPE pretreatment (25 and 50 mg/kg) before radiation exposure did not improve sperm abnormality in the IR group (*p* > 0.9999 and *p* = 0.6502, respectively), and the values of the CPE‐pretreated groups were slightly higher than those of the IR group. There was no significant difference between the CPE‐treated groups before irradiation (*p* > 0.9999). The sperm motility of the sham and CPE‐only treated groups was almost 100%, but the sperm motility was significantly damaged by irradiation exposure (5 Gy) (*p* < 0.0001; Figure 5b). Pretreatment with high‐dose CPE before irradiation significantly increased sperm motility compared with that in the IR group (*p* = 0.0135) and the low‐dose CPE‐pretreated group (*p* = 0.0062), although pretreatment with low‐dose CPE did not result in any change in radiation‐induced damage to sperm motility (*p* = 0.9308). Irradiation exposure (5 Gy) significantly decreased the number of sperm compared with that in the sham group (*p* < 0.0001; Figure 5c). There was a considerable improvement in sperm numbers in the CPE‐pretreated groups (25 and 50 mg/kg) before radiation compared to those in the IR group (*p* = 0.0316 and *p* = 0.0354, respectively), but there was no significant difference between the CPE‐treated groups before irradiation (*p* = 0.7033).

### 3.6 Antioxidative Potentials of CPE

**FIGURE 6:**
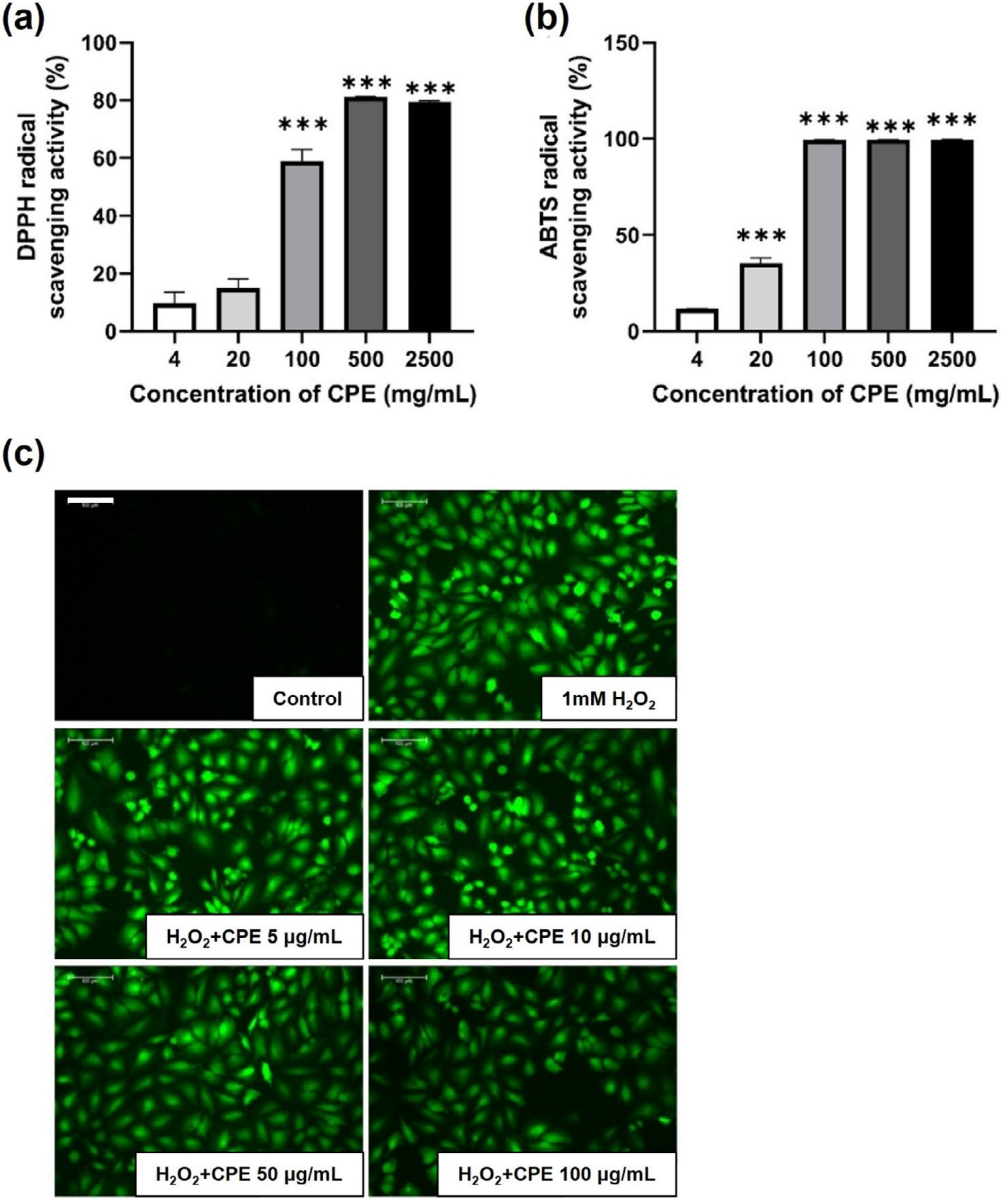
Antioxidative capacity of CPE in in vitro assay. Graphs showing the DPPH radical scavenging activity (%) (a) and ABTS radical scavenging activity (%) (b). Representative images (×400) showing ROS scavenging activity of CPE with various levels of DCFDA fluorescence intensity in HeLa cell (c). Data are reported as the mean ± SEM (*n* = four per group). ***p < 0.001 versus CPE 4 mg/mL‐treated group. Scale bar = 100 μm. CPE, water extract of *Cicadidae Periostracum*; DPPH, 2,2‐diphenyl‐1‐picrylhydrazyl; ABTS, 2,2′‐azinobis‐(3‐ethylbenzothiazoline‐6‐sulphonic acid).

To evaluate the effect of CPE treatment on ROS generation, we confirmed its antioxidative activity. CPE showed significantly higher antioxidative activities in DPPH (*p* = 0.1995 and *p* < 0.0001 for remaining comparisons)‐ and ABTS (*p* < 0.0001 for all comparisons)‐scavenging assays compared to those observed in the CPE 4 mg/mL‐treated group, in a dose‐dependent manner (20, 100, 500, and 2500 mg/mL); the half inhibitory concentration (IC50) was 83.49 and 42.71 mg/mL in DPPH and ABTS scavenging assays, respectively (Figure 6a,b). The inhibition rate plateaued at CPE concentrations of 500 and 100 mg/mL in the DPPH and ABTS assays, respectively.

Furthermore, we examined the antioxidant effects of CPE using a DCFDA assay (Figure 6c). No fluorescence was detected in the cells before H_2_O_2_ treatment. H_2_O_2_ treatment significantly increased the fluorescence intensity, indicating ROS production, whereas CPE treatment decreased the fluorescence intensity of H_2_O_2_‐treated cells in a dose‐dependent manner.

## 4 Discussion

The testis is a highly proliferative tissue with a rapid cellular renewal system and weak antioxidant defenses, making it a vulnerable target for radiation‐induced ROS damage (Sharma et al. 2015). The primary cause of radiation‐induced reproductive cell death is DNA damage induced by ROS, which can also promote mutations, germ cell apoptosis, and the development of chronic testicular atrophy, leading to long‐lasting azoospermia and infertility (Aitken and Krausz 2001; Kemal Duru et al. 2000). Free radicals cannot be completely neutralized by the intrinsic antioxidant system of the body. The use of antioxidants and antioxidant therapy can terminate or delay the oxidative chain reaction, significantly improve the ability of the body to combat free radical‐induced ROS, especially in the testes, and enhance spermatogenesis (Asadi et al. 2017). Therefore, various efforts have been made to ameliorate radiation‐induced oxidative damage to testis tissue, including research on the development of supplementary materials, such as selenium nanoparticles and *Lactobacillus casei* as probiotic components (Ehghaghi et al. 2022), monophosphoryl lipid A (Liu et al. 2020), dimethyl sulfoxide (Huang, Wang, et al. 2022; Huang, Peng, et al. 2022), and the natural products *Tinospora cordifolia* (Sharma et al. 2011), a*loe vera* (Gehlot et al. 2007), and *Protaetia brevitarsis seulensis* larvae, which we have previously studied (Nam et al. 2022). These papers have shown the radioprotective effects of the candidate substances based on their antioxidative effects on several testicular functional indexes, such as the number and morphology of cell populations (spermatogonia, spermatid, sperm, and Leydig's cell), the testis architecture (diameter and epithelial height of seminiferous tubule), or sperm motility.

In this study, we investigated the effect of CPE pretreatment on radiation‐induced damage to the testes as a potential natural product candidate. TUNEL staining was performed to evaluate oxidative damage through apoptotic nuclei expression (Haeri et al. 2014). Apoptotic nuclei increased in the irradiated group compared with the sham group, which is consistent with previous findings (Shimizu et al. 2011). In our study, CPE pretreatment ameliorated apoptotic damage dose‐dependently in irradiated tissues. Ionizing radiation can induce structural impairment and damage to testicular tissues (De Felice et al. 2019). We also found that the epithelial height and diameter of seminiferous tubules in the testes and the weights of related tissues were decreased by radiation exposure. However, CPE pretreatment mitigated the radiation‐induced structural damage and atrophy in the testes and epididymis. The diagnosis of sperm pathology has been used to evaluate sperm quality and fertility outcomes (Chemes and Rawe 2003). Sperm count is reduced by radiotherapy (Meistrich 1993). Furthermore, radiation (3.5–6 Sv) can induce permanent infertility and congenital anomalies by damaging male gonads (Wdowiak et al. 2019). In the present study, exposure to 5 Gy of radiation significantly influenced sperm characteristics, including sperm abnormality, motility, and number, whereas CPE pretreatment had a protective effect on sperm motility and number.

Previous studies confirmed the bioactive effects of CPE in several in vitro and in vivo models, including oxidative stress states. Chang et al. showed that CPE exerted antioxidant effects against ultraviolet B through nuclear factor E2‐related factor 2 (Nrf2) signaling in HaCaT keratinocytes (Chang et al. 2017). Zhang et al. reported that CP showed antioxidative and anti‐apoptotic effects, possibly via the phosphoinositide 3‐kinase/protein kinase B/Nrf2 signaling pathway, in H_2_O_2_‐induced PC12 cells (Zhang et al. 2021). Lin et al. reported that the ethyl acetate extract of CP decreased the expression of pro‐inflammatory cytokines and surface markers in lipopolysaccharide‐stimulated dendritic cells (Lin et al. 2025). Furthermore, several studies have identified the antioxidant, anti‐inflammatory, anti‐apoptotic, and antiepileptic effects of CPE in animal models of 1‐methyl‐4‐phenyl‐1,2,3,6‐tetrahydropyridine‐induced Parkinson's disease (Lim et al. 2019), pentylenetetrazole‐induced epilepsy (Zhang et al. 2021), dinitrochlorobenzene‐induced (Park et al. 2023), and house dust mite‐induced atopic dermatitis (Park et al. 2021). Here, we confirmed the antioxidative capacity of CPE using DPPH and ABTS radical scavenging assays, as well as a DCFDA assay, analyzed the chemical constituents of CPE using HPLC‐MS, and isolated two *N*‐acetyl dopamine dimers, (2*R*,3*S*)‐2‐(3′,4′‐dihydroxyphenyl)‐3‐acetylamino‐7‐(*N*‐acetyl‐2″‐aminoethyl)‐1,4‐benzodioxane and (2*R*,3*S*)‐2‐(3′,4′‐dihydroxyphenyl)‐3‐acetylamino‐6‐(*N*‐acetyl‐2″‐aminoethyl)‐1,4‐benzodioxane, previously reported in the extract of CP (Cao et al. 2020; Xu et al. 2006). *N*‐acetyl dopamine derivatives have been identified in insect species and are considered as sclerotizing agents of the insect cuticle (Karlson and Sekeris 1962). Several studies have investigated the bioactivity of two *N*‐acetyl dopamine dimers, (**1**) and (**2**). Xu et al. and Liu et al. demonstrated the antioxidant effects of compound (**1**) isolated from *Periostracum Cicadae* (Xu et al. 2006) and adult *Vespa velutina auraria* Smith (Liu et al. 2024), respectively. Yan et al. and Huang et al. reported the anti‐inflammatory effects of compound (**1**) or compound (**2**) isolated from *Blaps japanensis* (Yan et al. 2015) and *Isaria cicadae* (Huang, Wang, et al. 2022; Huang, Peng, et al. 2022), respectively. In the present study, we did not confirm the antioxidative effect of each component. Nevertheless, we isolated two *N*‐acetyl dopamine dimers as main components of CPE, and there are several studies about the bioactivities of the chemical constituents. Based on previous studies and the present results, we assumed that the protective effect of CPE against radiation‐induced testicular injury could be related to its antioxidant effects.

In conclusion, this study demonstrated that CPE pretreatment ameliorated testicular tissue damage and sperm degradation caused by irradiation in mice; the protective effects may be attributed to the antioxidative capacity of CPE. Therefore, we suggest that CPE might be a potential candidate for treating testicular injury induced by radiotherapy. As a limitation of the present study, we could not investigate the protective effect of each compound of CPE in vivo; thus, further investigation is needed to elucidate which compound mainly contributes to mitigating radiation‐induced testicular oxidative stress.

## Author Contributions

**Yun‐Soo Seo:** data curation (equal), formal analysis (equal), investigation (lead), writing – original draft (equal). **Seung Mok Ryu:** data curation (equal), investigation (equal), writing – original draft (equal). **Jun Lee:** investigation (equal). **Huiyeong Jeong:** investigation (equal). **Goya Choi:** funding acquisition (equal), project administration (equal). **Byeong Cheol Moon:** funding acquisition (equal). **Je‐Oh Lim:** investigation (equal). **Hyeon‐Hwa Nam:** investigation (equal). **Joong‐Sun Kim:** conceptualization (lead), data curation (equal), investigation (equal), validation (lead), writing – review and editing (equal). **Sueun Lee:** data curation (equal), investigation (equal), visualization (lead), writing – original draft (lead), writing – review and editing (lead).

## Ethics Statement

All procedures were performed with the approval of the Institutional Animal Care and Use Committee of the Korea Institute of Oriental Medicine (KIOM 20‐002, January 2020).

## Conflicts of Interest

The authors declare no conflicts of interest.

## Data Availability

The datasets used and/or analyzed during the current study are available from the corresponding author upon reasonable request.

## References

[fsn370198-bib-0001] Aitken, R. J. , and C. Krausz . 2001. “Oxidative Stress, DNA Damage and the Y Chromosome.” Reproduction 122, no. 4: 497–506. 10.1530/rep.0.1220497. 11570956

[fsn370198-bib-0002] Asadi, N. , M. Bahmani , A. Kheradmand , and M. Rafieian‐Kopaei . 2017. “The Impact of Oxidative Stress on Testicular Function and the Role of Antioxidants in Improving It: A Review.” Journal of Clinical and Diagnostic Research 11, no. 5: Ie01–Ie05. 10.7860/jcdr/2017/23927.9886. 28658802 PMC5483704

[fsn370198-bib-0003] Bentzen, S. M. 2006. “Preventing or Reducing Late Side Effects of Radiation Therapy: Radiobiology Meets Molecular Pathology.” Nature Reviews. Cancer 6, no. 9: 702–713. 10.1038/nrc1950. 16929324

[fsn370198-bib-0004] Berkey, F. J. 2010. “Managing the Adverse Effects of Radiation Therapy.” American Family Physician 82, no. 4: 381–388, 394. 20704169

[fsn370198-bib-0005] Borek, C. 2004. “Antioxidants and Radiation Therapy.” Journal of Nutrition 134, no. 11: 3207s–3209s. 10.1093/jn/134.11.3207S. 15514309

[fsn370198-bib-0006] Cao, X. C. , X. Y. Zhang , J. D. Xu , et al. 2020. “Quality Consistency Evaluation on Four Origins of Cicadae Periostracum by Ultra‐Performance Liquid Chromatography Coupled With Quadrupole/Time‐of‐Flight Mass Spectrometry Analysis.” Journal of Pharmaceutical and Biomedical Analysis 179: 112974. 10.1016/j.jpba.2019.112974. 31767224

[fsn370198-bib-0007] Chang, T. M. , J. H. Tsen , H. Yen , T. Y. Yang , and H. C. Huang . 2017. “Extract From Periostracum Cicadae Inhibits Oxidative Stress and Inflammation Induced by Ultraviolet B Irradiation on HaCaT Keratinocytes.” Evidence‐Based Complementary and Alternative Medicine 2017: 8325049. 10.1155/2017/8325049. 28465707 PMC5390570

[fsn370198-bib-0008] Chemes, E. H. , and Y. V. Rawe . 2003. “Sperm Pathology: A Step Beyond Descriptive Morphology. Origin, Characterization and Fertility Potential of Abnormal Sperm Phenotypes in Infertile Men.” Human Reproduction Update 9, no. 5: 405–428. 10.1093/humupd/dmg034. 14640375

[fsn370198-bib-0009] De Felice, F. , C. Marchetti , F. Marampon , G. Cascialli , L. Muzii , and V. Tombolini . 2019. “Radiation Effects on Male Fertility.” Andrology 7, no. 1: 2–7. 10.1111/andr.12562. 30411532

[fsn370198-bib-0010] Dillon, K. E. , and C. R. Gracia . 2012. “Pediatric and Young Adult Patients and Oncofertility.” Current Treatment Options in Oncology 13, no. 2: 161–173. 10.1007/s11864-012-0183-7. 22422325

[fsn370198-bib-0011] Ehghaghi, A. , E. Zokaei , M. H. Modarressi , et al. 2022. “Antioxidant and Anti‐Apoptotic Effects of Selenium Nanoparticles and *Lactobacillus casei* on Mice Testis After X‐Ray.” Andrologia 54, no. 11: e14591. 10.1111/and.14591. 36266770

[fsn370198-bib-0012] Gehlot, P. , D. Soyal , and P. Goyal . 2007. “Alterations in Oxidative Stress in Testes of Swiss Albino Mice by Aloe Vera Leaf Extract After Gamma Irradiation.” Pharmacology 1: 359–370.

[fsn370198-bib-0013] Haeri, S. A. , H. Rajabi , S. Fazelipour , and S. J. Hosseinimehr . 2014. “Carnosine Mitigates Apoptosis and Protects Testicular Seminiferous Tubules From Gamma‐Radiation‐Induced Injury in Mice.” Andrologia 46, no. 9: 1041–1046. 10.1111/and.12193. 24215656

[fsn370198-bib-0015] Huang, Z. , R. Peng , H. Yu , et al. 2022. “Dimethyl Sulfoxide Attenuates Radiation‐Induced Testicular Injury Through Facilitating DNA Double‐Strand Break Repair.” Oxidative Medicine and Cellular Longevity 2022: 9137812. 10.1155/2022/9137812. 35770047 PMC9236762

[fsn370198-bib-0014] Huang, L. J. , Y. M. Wang , L. Q. Gong , et al. 2022. “N‐Acetyldopamine Dimer Attenuates DSS‐Induced Ulcerative Colitis by Suppressing NF‐κB and MAPK Pathways.” Frontiers in Pharmacology 13: 842730. 10.3389/fphar.2022.842730. 35462925 PMC9030057

[fsn370198-bib-0016] Karlson, P. , and C. E. Sekeris . 1962. “N‐Acetyl‐Dopamine as Sclerotizing Agent of the Insect Cuticle.” Nature 195, no. 4837: 183–184.

[fsn370198-bib-0017] Kemal Duru, N. , M. Morshedi , and S. Oehninger . 2000. “Effects of Hydrogen Peroxide on DNA and Plasma Membrane Integrity of Human Spermatozoa.” Fertility and Sterility 74, no. 6: 1200–1207. 10.1016/s0015-0282(00)01591-0. 11119751

[fsn370198-bib-0018] Kim, J. , S. Lee , B. Jeon , W. Jang , C. Moon , and S. Kim . 2011. “Protection of Spermatogenesis Against Gamma Ray‐Induced Damage by Granulocyte Colony‐Stimulating Factor in Mice.” Andrologia 43, no. 2: 87–93. 10.1111/j.1439-0272.2009.01023.x. 21382061

[fsn370198-bib-0019] Kim, J. S. , K. Heo , J. M. Yi , et al. 2012. “Genistein Mitigates Radiation‐Induced Testicular Injury.” Phytotherapy Research 26, no. 8: 1119–1125. 10.1002/ptr.3689. 22162311

[fsn370198-bib-0020] Kovacs, G. T. , and K. Stern . 1999. “Reproductive Aspects of Cancer Treatment: An Update.” Medical Journal of Australia 170, no. 10: 495–497. 10.5694/j.1326-5377.1999.tb127853.x. 10376028

[fsn370198-bib-0021] Lee, H. J. , J. S. Kim , C. Moon , J. C. Kim , S. K. Jo , and S. H. Kim . 2008. “Relative Biological Effectiveness of Fast Neutrons in a Multiorgan Assay for Apoptosis in Mouse.” Environmental Toxicology 23, no. 2: 233–239. 10.1002/tox.20328. 18214905

[fsn370198-bib-0022] Lee, W. , H. Lee , M. A. Kim , et al. 2017. “Evaluation of Novel Factor Xa Inhibitors From Oxya Chinensis Sinuosa With Anti‐Platelet Aggregation Activity.” Scientific Reports 7, no. 1: 7934. 10.1038/s41598-017-08330-1. 28801633 PMC5554137

[fsn370198-bib-0023] Lim, H. S. , J. S. Kim , B. C. Moon , et al. 2019. “Cicadidae Periostracum, the Cast‐Off Skin of Cicada, Protects Dopaminergic Neurons in a Model of Parkinson's Disease.” Oxidative Medicine and Cellular Longevity 2019: 5797512. 10.1155/2019/5797512. 31772707 PMC6854990

[fsn370198-bib-0024] Lin, W. H. , C. H. Chuang , P. W. Chen , et al. 2025. “Periostracum Cicadae Exhibits Immunosuppressive Effects on Dendritic Cells and Contact Hypersensitivity Responses.” Journal of Ethnopharmacology 337, no. Pt 1: 118824. 10.1016/j.jep.2024.118824. 39270880

[fsn370198-bib-0025] Liu, C. H. , X. Q. Pang , Q. Yu , et al. 2024. “Anti‐Inflammatory and Antioxidative N‐Acetyldopamine Dimers From Adult Vespa Velutina Auraria Smith.” Molecules 29, no. 22: 5445. 10.3390/molecules29225445. 39598834 PMC11597435

[fsn370198-bib-0026] Liu, Z. , K. Cao , Z. Liao , et al. 2020. “Monophosphoryl Lipid A Alleviated Radiation‐Induced Testicular Injury Through TLR4‐Dependent Exosomes.” Journal of Cellular and Molecular Medicine 24, no. 7: 3917–3930. 10.1111/jcmm.14978. 32135028 PMC7171420

[fsn370198-bib-0027] Luo, J. , W. Wei , P. Wang , et al. 2022. “(±)‐Cryptamides A‐D, Four Pairs of Novel Dopamine Enantiomer Trimers From the Periostracum Cicadae.” Molecules 27, no. 19: 6707. 10.3390/molecules27196707. 36235243 PMC9571589

[fsn370198-bib-0028] Majeed, H. , and V. Gupta . 2025. “Adverse Effects of Radiation Therapy.” In StatPearls. StatPearls Publishing LLC. 33085406

[fsn370198-bib-0029] Meistrich, M. L. 1993. “Effects of Chemotherapy and Radiotherapy on Spermatogenesis.” European Urology 23, no. 1: 136–141; discussion 142. 8477773

[fsn370198-bib-0030] Nam, H. H. , S. Kang , Y. S. Seo , et al. 2022. “Protective Effects of an Aqueous Extract of Protaetia Brevitarsis Seulensis Larvae Against Radiation‐Induced Testicular Injury in Mice.” Food Science & Nutrition 10, no. 11: 3969–3978. 10.1002/fsn3.2992. 36348800 PMC9632216

[fsn370198-bib-0031] Nam, J. H. , H. W. Jung , W. K. Kim , and H. S. Bae . 2018. “Modulatory Effect of Periostracum Cicadae and Betulae Cortex Extracts on the Activation of Atopic Dermatitis‐Related Ion Channels orai1 and TRPV3.” African Journal of Traditional, Complementary, and Alternative Medicines 15, no. 1: 183–187. 10.21010/ajtcam.v15i1.19.

[fsn370198-bib-0032] Oakberg, E. F. 1956. “Duration of Spermatogenesis in the Mouse and Timing of Stages of the Cycle of the Seminiferous Epithelium.” American Journal of Anatomy 99, no. 3: 507–516. 10.1002/aja.1000990307. 13402729

[fsn370198-bib-0033] Okada, K. , and M. Fujisawa . 2019. “Recovery of Spermatogenesis Following Cancer Treatment With Cytotoxic Chemotherapy and Radiotherapy.” World Journal of Men's Health 37, no. 2: 166–174. 10.5534/wjmh.180043. PMC6479085 30588779

[fsn370198-bib-0034] Park, G. , N. Kwon , M. H. Kim , and W. M. Yang . 2023. “The Slough of Cicadidae Periostracum Ameliorated Lichenification by Inhibiting Interleukin (IL)‐22/Janus Kinase (JAK) 1/Signal Transducer and Activator of Transcription (STAT) 3 Pathway in Atopic Dermatitis.” Food Science of Animal Resources 43, no. 5: 859–876. 10.5851/kosfa.2023.e40. 37701738 PMC10493567

[fsn370198-bib-0035] Park, G. , B. C. Moon , S. M. Ryu , W. J. Kim , and H. S. Lim . 2021. “Cicadidae Periostracum Attenuates Atopic Dermatitis Symptoms and Pathology via the Regulation of NLRP3 Inflammasome Activation.” Oxidative Medicine and Cellular Longevity 2021: 8878153. 10.1155/2021/8878153. 33520088 PMC7817262

[fsn370198-bib-0036] Sharma, P. , J. Parmar , P. Sharma , P. Verma , and P. K. Goyal . 2011. “Radiation‐Induced Testicular Injury and Its Amelioration by Tinospora Cordifolia (An Indian Medicinal Plant) Extract.” Evidence‐Based Complementary and Alternative Medicine 2011: 643847. 10.1155/2011/643847. 21350610 PMC3042631

[fsn370198-bib-0037] Sharma, P. , J. Parmar , P. Verma , and P. Goyal . 2015. “Radiation Induced Oxidative Stress and Its Toxicity in Testes of Mice and Their Prevention by Tinospora Cordifolia Extract.” Journal of Reproductive Health and Medicine 1, no. 2: 64–75. 10.1016/j.jrhm.2015.01.005.

[fsn370198-bib-0038] Shimizu, S. , M. Saito , F. Dimitriadis , et al. 2011. “Protective Effect of Ischaemic Post‐Conditioning on Ipsilateral and Contralateral Testes After Unilateral Testicular Ischaemia‐Reperfusion Injury.” International Journal of Andrology 34, no. 3: 268–275. 10.1111/j.1365-2605.2010.01077.x. 20522123

[fsn370198-bib-0039] Thapa, P. , N. Katila , D.‐Y. Choi , H. Choi , and J.‐W. Nam . 2021. “Suntamide A, a neuroprotective cyclic peptide from Cicadidae Periostracum.” Bioorganic Chemistry 106: 104493. 10.1016/j.bioorg.2020.104493. 33268010

[fsn370198-bib-0040] Thapa, P. , N. Katila , H. Choi , A.‐R. Han , D.‐Y. Choi , and J.‐W. Nam . 2021. “Neuroprotective Effects of n‐Acetyldopamine Dimers From Cicadidae Periostracum.” Natural Product Sciences 27, no. 3: 161–168.

[fsn370198-bib-0041] Tiwari, R. , M. H. Siddiqui , T. Mahmood , et al. 2020. “An Exploratory Analysis on the Toxicity & Safety Profile of Polyherbal Combination of Curcumin, Quercetin and Rutin.” Clinical Phytoscience 6, no. 1: 1–18. 10.1186/s40816-020-00228-2.

[fsn370198-bib-0042] Wang, J. , Q. Tian , G. Tao , Q. Gao , T. Lv , and D. Wang . 2010. “Analyses on Ingredients and Antibacterial Activity of Periostracum Cicadae.” Chinese Bulletin of Entomology 47, no. 6: 1109–1112.

[fsn370198-bib-0043] Wdowiak, A. , M. Skrzypek , M. Stec , and L. Panasiuk . 2019. “Effect of Ionizing Radiation on the Male Reproductive System.” Annals of Agricultural and Environmental Medicine 26, no. 2: 210–216. 10.26444/aaem/106085. 31232047

[fsn370198-bib-0044] Xu, M. Z. , W. S. Lee , J. M. Han , et al. 2006. “Antioxidant and Anti‐Inflammatory Activities of N‐Acetyldopamine Dimers From Periostracum Cicadae.” Bioorganic and Medicinal Chemistry 14, no. 23: 7826–7834. 10.1016/j.bmc.2006.07.063. 16919462

[fsn370198-bib-0045] Yan, Y. M. , L. J. Li , X. C. Qin , Q. Lu , Z. C. Tu , and Y. X. Cheng . 2015. “Compounds From the Insect Blaps Japanensis With COX‐1 and COX‐2 Inhibitory Activities.” Bioorganic and Medicinal Chemistry Letters 25, no. 12: 2469–2472. 10.1016/j.bmcl.2015.04.085. 25980909

[fsn370198-bib-0046] Zhang, Q. , R. L. Li , T. Tao , et al. 2021. “Antiepileptic Effects of Cicadae Periostracum on Mice and Its Antiapoptotic Effects in H_2_O_2_‐stimulated PC12 Cells via Regulation of PI3K/Akt/Nrf2 Signaling Pathways.” Oxidative Medicine and Cellular Longevity 2021: 5598818. 10.1155/2021/5598818. 34336105 PMC8324375

